# Checklist of the fish fauna of the Munim River Basin, Maranhão, north-eastern Brazil

**DOI:** 10.3897/BDJ.11.e98632

**Published:** 2023-02-10

**Authors:** Lucas O. Vieira, Diego S. Campos, Rafael F. Oliveira, Josie South, Marcony S. P. Coelho, Maurício J. S. Paiva, Pedro H. N. Bragança, Erick C. Guimarães, Axel M. Katz, Pâmella S. Brito, Jadson P. Santos, Felipe P. Ottoni

**Affiliations:** 1 Universidade Federal do Maranhão, Centro de Ciências de Chapadinha, Campus de Chapadinha, Programa de Pós-Graduação em Ciências Ambientais, BR-222, KM 04, Boa Vista, CEP: 65500-000, Chapadinha, Brazil Universidade Federal do Maranhão, Centro de Ciências de Chapadinha, Campus de Chapadinha, Programa de Pós-Graduação em Ciências Ambientais, BR-222, KM 04, Boa Vista, CEP: 65500-000 Chapadinha Brazil; 2 Universidade Federal do Maranhão, Centro de Ciências de Chapadinha, Campus de Chapadinha, Laboratório de Sistemática e Ecologia de Organismos Aquáticos, BR-222, KM 04, S/N, Boa Vista, CEP: 65500-000, Chapadinha, Brazil Universidade Federal do Maranhão, Centro de Ciências de Chapadinha, Campus de Chapadinha, Laboratório de Sistemática e Ecologia de Organismos Aquáticos, BR-222, KM 04, S/N, Boa Vista, CEP: 65500-000 Chapadinha Brazil; 3 Universidade Federal do Maranhão, Programa de Pós-Graduação em Biodiversidade e Biotecnologia da Amazônia Legal, Av. dos Portugueses, 1966, Cidade Universitária Dom Delgado, 65080-805, São Luís, Brazil Universidade Federal do Maranhão, Programa de Pós-Graduação em Biodiversidade e Biotecnologia da Amazônia Legal, Av. dos Portugueses, 1966, Cidade Universitária Dom Delgado, 65080-805 São Luís Brazil; 4 Universidade Federal do Maranhão, Programa de Pós-Graduação em Biodiversidade e Conservação, Av. dos Portugueses, 1966, CEP: 65085-580, São Luís, Brazil Universidade Federal do Maranhão, Programa de Pós-Graduação em Biodiversidade e Conservação, Av. dos Portugueses, 1966, CEP: 65085-580 São Luís Brazil; 5 School of Biology, Faculty of Biological Sciences, University of Leeds, Leeds, United Kingdom School of Biology, Faculty of Biological Sciences, University of Leeds Leeds United Kingdom; 6 South African Institute for Aquatic Biodiversity, Grahamstown, South Africa South African Institute for Aquatic Biodiversity Grahamstown South Africa; 7 Universidade Federal do Maranhão, Departamento de Biologia, Laboratório de Genética e Biologia Molecular, Av. dos Portugueses 1966, Cidade Universitária do Bacanga, CEP: 65080-805, São Luís, Brazil Universidade Federal do Maranhão, Departamento de Biologia, Laboratório de Genética e Biologia Molecular, Av. dos Portugueses 1966, Cidade Universitária do Bacanga, CEP: 65080-805 São Luís Brazil; 8 Universidade Estadual do Maranhão, Laboratório de Ictiofauna e Piscicultura Integrada, Centro de Ciências Agrárias, Campus Paulo VI, avenida Lourenço Vieira da Silva, n. 1000, bairro Jardim São Cristóvão, CEP: 65.055-310, São Luís, Brazil Universidade Estadual do Maranhão, Laboratório de Ictiofauna e Piscicultura Integrada, Centro de Ciências Agrárias, Campus Paulo VI, avenida Lourenço Vieira da Silva, n. 1000, bairro Jardim São Cristóvão, CEP: 65.055-310 São Luís Brazil; 9 Universidade Federal do Oeste do Pará, Instituto de Ciências da Educação, Programa de Pós-graduação Sociedade Natureza e Desenvolvimento, Av. Marechal Rondon s/n, CEP: 68040-070, Santarém, PA, Brasil, Belém, Brazil Universidade Federal do Oeste do Pará, Instituto de Ciências da Educação, Programa de Pós-graduação Sociedade Natureza e Desenvolvimento, Av. Marechal Rondon s/n, CEP: 68040-070, Santarém, PA, Brasil Belém Brazil; 10 Universidade Federal do Rio de Janeiro, Laboratório de Sistemática e Evolução de Peixes Teleósteos, Programa de Pós-Graduação em Biodiversidade e Biologia Evolutiva, Instituto de Biologia, CEP: 21.941-902, Rio de Janeiro, Brazil Universidade Federal do Rio de Janeiro, Laboratório de Sistemática e Evolução de Peixes Teleósteos, Programa de Pós-Graduação em Biodiversidade e Biologia Evolutiva, Instituto de Biologia, CEP: 21.941-902 Rio de Janeiro Brazil

**Keywords:** biodiversity, endemism, freshwater, migratory species, taxonomy.

## Abstract

**Background:**

The Maranhão State harbours great fish diversity, but some areas are still undersampled or little known, such as the Munim River Basin in the northeast of the State. This lack of knowledge is critical when considering anthropogenic impacts on riverine systems especially in the face of major habitat destruction. These pressing threats mean that a comprehensive understanding of diversity is critical and fish checklists extremely relevant. Therefore, the present study provides a checklist of the fish species found in the Munim River Basin, Maranhão State, north-eastern Brazil, based on collected specimens.

**New information:**

A total of 123 species were recorded for the Munim River Basin, with only two non-native species, *Oreochromisniloticus* and *Colossomamacropomum*, showing that the fish assemblage has relatively high ecological integrity. In addition, 29 species could not be identified at the species level, indicating the presence of species that are probably new to science in the Basin. A predominance of species belonging to the fish orders Characiformes and Siluriformes, with Characidae being recovered as the most species-rich family (21 species) agrees with the general pattern for river basins in the Neotropical Region. The total fish diversity was estimated by extensive fieldwork, including several sampling gears, carried out in different seasons (dry and rainy) and exploring different environments with both daily and nocturnal sampling, from the Basin's source to its mouth. A total of 84 sites were sampled between 2010 and 2022, resulting in 12 years of fieldwork. Fish assemblages were distinct in the Estuary and Upper river basin sections and more similar in the Lower and Middle sections indicating environmental filtering processes. Species were weakly nested across basin sections, but unique species were found in each section (per Simpsons Index). High variability of species richness in the Middle river basin section is likely due to microhabitat heterogeneity supporting specialist fish communities.

## Introduction

The Neotropical Region comprises the most biodiverse freshwater ichthyofauna on the planet, with more than 6000 described species (Reis et al. 2016, Albert et al. 2020). Within the Neotropics, South America harbours the world's greatest diversity of freshwater fishes, including about 5160 described species, which represents about one-third of all known freshwater species (Reis et al. 2016, Pelicice et al. 2017, Castro and Polaz 2020). Studies on diversity of the region have produced estimates which are much higher, predictions being between 8000 to 9000 described and undescribed freshwater fish species (Reis et al. 2016, Birindelli and Sidlauskas 2018, Castro and Polaz 2020, Albert et al. 2020, Koerber et al. 2022). This high diversity is mainly comprised of medium- to small-sized species (species that do not surpass 15 cm standard length), corresponding to 70% of the species (Reis et al. 2003, Castro and Polaz 2020). Small and medium-sized species are broadly distributed throughout all aquatic habitats, which is most likely due to niche partitioning, life history traits adapted to stochastic environments and high trophic plasticity (Vazzoler 1996, Castro 1999, Lowe-Mcconnell 1999, Abelha et al. 2001, Guimarães et al. 2020, Castro and Polaz 2020, Corrêa and Castro 2021). Despite the description of small- and medium-sized fish diversity in scientific journals, they remain largely unnoticed by the general public and neglected by conservation agencies and policies (Castro 1999, Castro et al. 2005, Abell et al. 2011, Albert et al. 2011, Castro and Polaz 2020).

Brazil possesses the highest number of freshwater fish species in South America (Buckup et al. 2007, Castro and Polaz 2020), with about 100 new species being described every year over the last decade (Nelson et al. 2016, Reis et al. 2016, Fricke et al. 2022). However, several of these species represent endemics, with narrow distributions and some are highly threatened due to increased anthropogenic pressure on their natural habitats (Reis et al. 2003, Nogueira et al. 2010, Darwall et al. 2018, Reid et al. 2019). Brazilian freshwaters are subject to multitude anthropogenic threats, such as: deforestation resulting in suppression or reduction of the original vegetation cover, due to logging and expansion of agricultural and urban areas; release of domestic and industrial effluents and chemical products from agricultural activities in aquatic environments, resulting in pollution; irregular water abstraction for different urban, industrial and agricultural uses; soil erosion and silting of the environments; river damming and construction of hydroelectric power plants, disrupting fish migration routes and destroying the natural habitats of fish species; extraction of sand from the riverbeds; mining, resulting in modification of habitats and water pollution and contamination; modification and diversion of the river channels; introduction of non-native species; overharvesting for the aquarium trade; ghost fishing; and overfishing of food fishes (Dudgeon et al. 2006, Pereira et al. 2016, Pelicice et al. 2017, Reid et al. 2019, Zarfl et al. 2019, Zeni et al. 2019, Bergmann et al. 2020, Castro and Polaz 2020, Ottoni et al. 2021, Azevedo-Santos et al. 2021, Doria et al. 2021, Vitorino et al. 2022, Rocha et al. 2023). Despite the high freshwater native fish diversity, non-native fish species have proliferated in Brazil and in Brazilian hydrographic systems where they do not occur naturally due to several human activities, such as: aquaculture, intentional introductions and release, aquarium trade, mosquito larvae biological control interventions, transposition of water between isolated river basins, sport fishing, amongst other activities (Figueredo and Giani 2005, Azevedo-Santos et al. 2011, Vitule et al. 2015, Latini et al. 2016, Padial et al. 2017, Bragança et al. 2020, Doria et al. 2021, Ottoni et al. 2021, Franco et al. 2022, Rocha et al. 2023). Non-native species have caused changes in the local assemblage composition and in the abundance of native species populations, causing major environmental impacts (Giacomini et al. 2011, Latini et al. 2016, Padial et al. 2017, Doria et al. 2021, Ottoni et al. 2021, Rocha et al. 2023).

Maranhão is the westernmost state in north-eastern Brazil, bordered by the Piauí State in the east, from whom it is separated by the Parnaíba River; by Tocantins State in the south and southeast, from which it is separated by the Tocantins River; and by Pará State in the west, from which it is separated by the Gurupi River (Rebêlo et al. 2003). Maranhão total area is about 330000 km^2^, corresponding to 3.9% of Brazil's territory (Rebêlo et al. 2003, Rios 2005, Batistella et al. 2014, Spinelli-Araújo et al. 2016). Maranhão is an extremely important State in terms of biodiversity, housing three of the main Brazilian biomes, as well as transition areas between them. The Cerrado biome is present in the central, eastern and southern portion of the State; the Amazon biome is present in the western and central portion; and the Caatinga biome is found in the easternmost portion of the State (Rebêlo et al. 2003, Rios 2005, Batistella et al. 2014, Spinelli-Araújo et al. 2016). Thus, Maranhão includes a phytogeographic mosaic due to the presence and overlap of floral elements typical of these three distinct biomes, besides the presence of complex transition areas, making the State extremely biodiverse, ecologically relevant and a key area for conservation (Rebêlo et al. 2003, Rios 2005, Batistella et al. 2014, Spinelli-Araújo et al. 2016).

In the past two decades, several fish surveys were carried out in Maranhão, in both freshwater and estuarine environments, increasing the knowledge of the State's fish fauna (Castro 2001, Castro et al. 2002, Piorski et al. 2003, Pinheiro-Júnior et al. 2005, Soares 2005, Piorski et al. 2007, Castro et al. 2010, Barros et al. 2011, Sousa et al. 2011, Fraga et al. 2012, Almeida et al. 2013, Ribeiro et al. 2014, Ramos et al. 2014, Lima et al. 2015, Matavelli et al. 2015, Melo et al. 2016, Nascimento et al. 2016, Piorski et al. 2017, Brito et al. 2019, Lima et al. 2019, Teixeira et al. 2019, Nunes et al. 2019, Guimarães et al. 2020, Brito et al. 2020, Oliveira et al. 2020, Guimarães et al. 2021c, Guimarães et al. 2021a, Guimarães et al. 2021b). Information about the ichthyofauna of the coastal Munim River Basin, however, is scarce. At the same time, this river basin is under severe anthropogenic pressure from deforestation of marginal vegetation, pollution, contamination of the water, erosion, siltation and even the loss of water bodies (Ribeiro et al. 2006, Ribeiro and Nunes 2017). The Munim River Basin has only five published studies documenting its fish diversity (Ribeiro et al. 2014, Matavelli et al. 2015, Nunes et al. 2019, Oliveira et al. 2020, Guimarães et al. 2021c). These, however, focused on specific localities and environments and, in many cases, surveying only similar and neighbouring sites within this river basin. As a consequence, the fish fauna of the Munim River Basin still awaits a more comprehensive checklist.

The main goal of the present study is to present a detailed inventory of the fish diversity in the Munim River Basin, through the analysis and study of data sampled over 12 years of fieldwork, providing species-level identifications when possible. The study covered the entire river basin and includes relevant information about the importance of checklists in contributing to the knowledge of the river basin, species conservation and distribution. In addition, we provide here ecological and biogeographical comments.

## Materials and methods

### Study area

**Figure 1.**
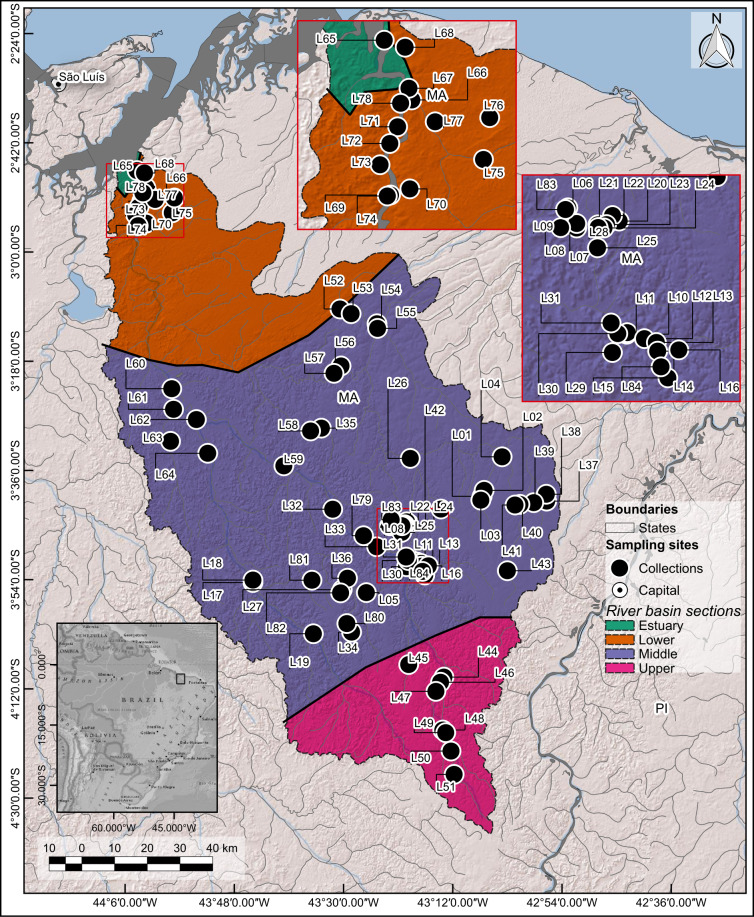
Map with sampling sites along the Munim River Basin. Sample sites are listed in Table 1 and illustrated on the map as L1-L84. MA = State of Maranhão and PI = State of Piauí. In the highlighted squares are the geographically close sample sites, for better visualisation. River basin sections: Estuary section (green), Lower section (orange), Middle section (lilac) and Upper section (pink).

Sampling was carried out in rivers, streams, lagoons, swamps, marshes, lakes and the estuary of the Munim River Basin, northeast of the Maranhão State, north-eastern Brazil. The Munim River Basin source is at the Caxias Municipality, in the Cerrado Biome and its mouth is at baía of São José in a region known as "Golfão Maranhense" between the Axixá and Icatu municipalities, within the Cerrado and Amazon biomes (Fig. 1). The Munim River Basin has an area of about 15918.04 km^2^, with 331.74 km from its source to its mouth (Nugeo 2016, Rios 2005).

### Sampling sites

**Figure 2.**
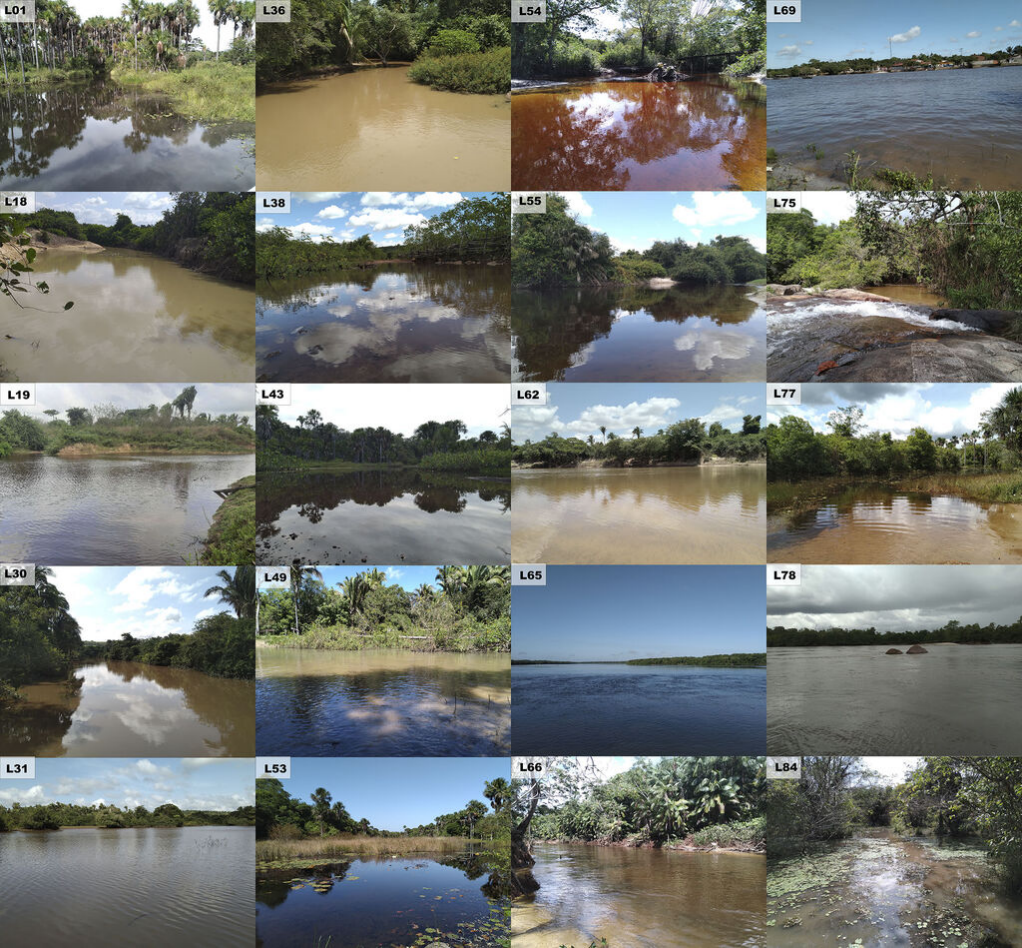
Samples sites: L1, L18, L19, L30, L31, L36, L38, L43, L49, L53, L54, L55, L62, L65, L66, L69, L75, L77, L78 and L84 according to Table 1. Photographed by Lucas Vieira and Rafael Oliveira, edited by Axel Katz.

Sampling was carried out in 84 collecting sites, covering four different sections of the Munim River Basin, in both rainy (January to May) and dry (June to December) seasons according to Passos et al. (2016). The sampling was done between 2010 and 2022 (about 65% of the surveys were carried out between 2019 and 2022), including sites close to its source and to its mouth (Fig. 1). The sampled environments included rivers, streams, lagoons, swamps, marshes, lakes and the estuary (Table 1, Fig. 2, Suppl. material 1).

### Sampling and specimens identification

All (about 160) sampling events were carried under the permits issued by Instituto Chico Mendes de Conservação da Biodiversidade (ICMBIO; License nº 54949, 57258, 57787, 64415, 73267). In addition, material already housed at the Coleção Ictiológica do Centro de Ciências Agrárias e Ambientais (CICCAA) of the Universidade Federal do Maranhão, was also used in this study. The specimens were sampled by using different sampling gear, such as fishing line, hand net, seine net, cast net, gill nets and crayfish-type traps (Souza and Auricchio 2002). All the sampling activities and procedures followed the best practices and standards for animal welfare as presented in Leary et al. (2020). Specimens were euthanised by immersion in a 250 mg/l Tricaine methane sulphonate (MS-222) solution until the cessation of opercular movements.

Following the euthanasia, the specimens for morphological studies were preserved in formalin (10%) and moved to a 70% ethanol solution after 10-15 days. Specimens selected for future molecular studies were preserved in 99% ethanol. The processing and identification of specimens were made at the Laboratório de Sistemática e Ecologia de Organismos Aquáticos (LASEOA), at the Universidade Federal do Maranhão, by the use of specialised bibliography for each taxonomic group and by consulting specialists. The specimens were identified to the lowest taxonomic rank possible. All biological material is catalogued and housed at the Coleção Ictiológica do Centro de Ciências Agrárias e Ambientais (CICCAA) of the Universidade Federal do Maranhão (UFMA) (Suppl. materials 1, 2). The taxonomic classifications, species names, authorship and year, original descriptions, habitat of occurrence and geographic distributions were verified and presented according to Fricke et al. (2022a), Fricke et al. (2022b) and Froese and Pauly (2022).

### Map and Munin River Basin sections distinction

The geographic coordinates of each collection site along the Munim River Basin were registered from a GPS device and then converted to the *shapefile* format, with place names and respective codes in the attribute table. Additional data on boundaries from river basins and political division of territory were acquired from the official data service IBGE (Brazilian Institute for Geography and Statistics). The map was composed in QGIS 3.22.12 (Qgis development team 2022). Due to scale, each point on the map may correspond to one or more collection sites, depending on the geographic proximity.

The Munim River Basin was divided into four sections: Estuary section with an area of 78.89 km^2^, comprising one collecting site; Lower river basin section with an area of 2891.89 km^2^, comprising 13 collecting sites; Middle river basin section with an area of 10722.29 km^2^, comprising 62 collecting sites; and Upper river basin section with an area of 2224.90 km^2^, comprising eight collecting sites (Fig. 1, Table 1, Suppl. material 2). The criterion for the sectorisation of the basin was based on the average slope calculated from the elevation values (meters above sea level) of the digital elevation model SRTM/USGS, available at the TOPODATA/INPE project (http://www.dsr.inpe.br/topodata/). Based on the analysed area, this river basin varies from 0 to 162 meters above the sea level. The parameters considered for the sectorisation were: Estuary section - average slope of 1.09 (standard deviation 1.59); Lower river basin section - average slope of 1.41 (standard deviation 1.33); Middle river basin section - average slope of 2.63 (standard deviation: 2.43); and Upper river basin section - average slope of 3.11 (standard deviation 2.61) (Fig. 1, Table 1, Suppl. material 2).

### Species photographs

**Figure 3.**
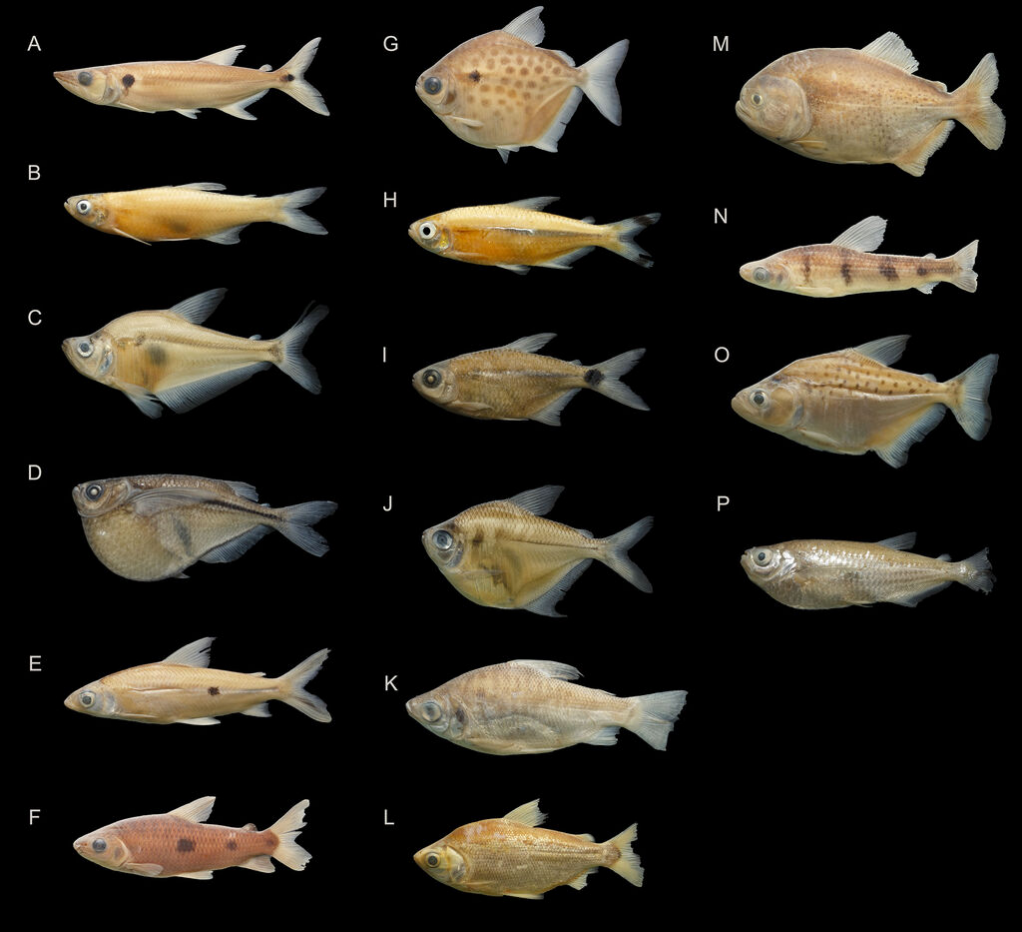
Selected fish species collected in the Munim River Basin of the Order Characiformes: **A** *Acestrorhynchusfalcatus* (CICCAA 06398, 112.60 mm SL), **B** *Aphyocharax* sp. (CICCAA 06636, 32.91 mm SL), **C** *Charaxawa* (CICCAA 06430, 80.22 mm SL), **D** *Gasteropelecussternicla* (CICCAA 06366, 39.50 mm SL), **E** *Hemiodusparnaguae* (CICCAA 06238, 94.99 mm SL), **F** Leporinusaff.friderici (CICCAA 02755, 102.31 mm SL), **G** *Metynnislippincottianus* (CICCAA 06383, 64.06 mm SL), **H** Moenkhausiacf.intermedia (CICCAA 06634, 50.38 mm SL), **I** *Moenkhausia* sp. (CICCAA 06635, 35.10 mm SL), **J** *Poptellacompressa* (CICCAA 06429, 42.46 mm SL), **K** *Prochiloduslacustris* (CICCAA 06340, 84.94 mm SL), **L** *Psectrogasterrhomboides* (CICCAA 06270, 121.08 mm SL), **M** *Pygocentrusnattereri* (CICCAA 06271, 138.08 mm SL), **N** *Schizodondissimilis* (CICCAA 06344, 99.03 mm SL), **O** *Serrasalmusrhombeus* (CICCAA 06269, 70.99 mm SL), **P** *Triportheussignatus* (CICCAA 06339, 86.62 mm SL). Photographed by Lucas Vieira and Rafael Oliveira, edited by Axel Katz.

**Figure 4.**
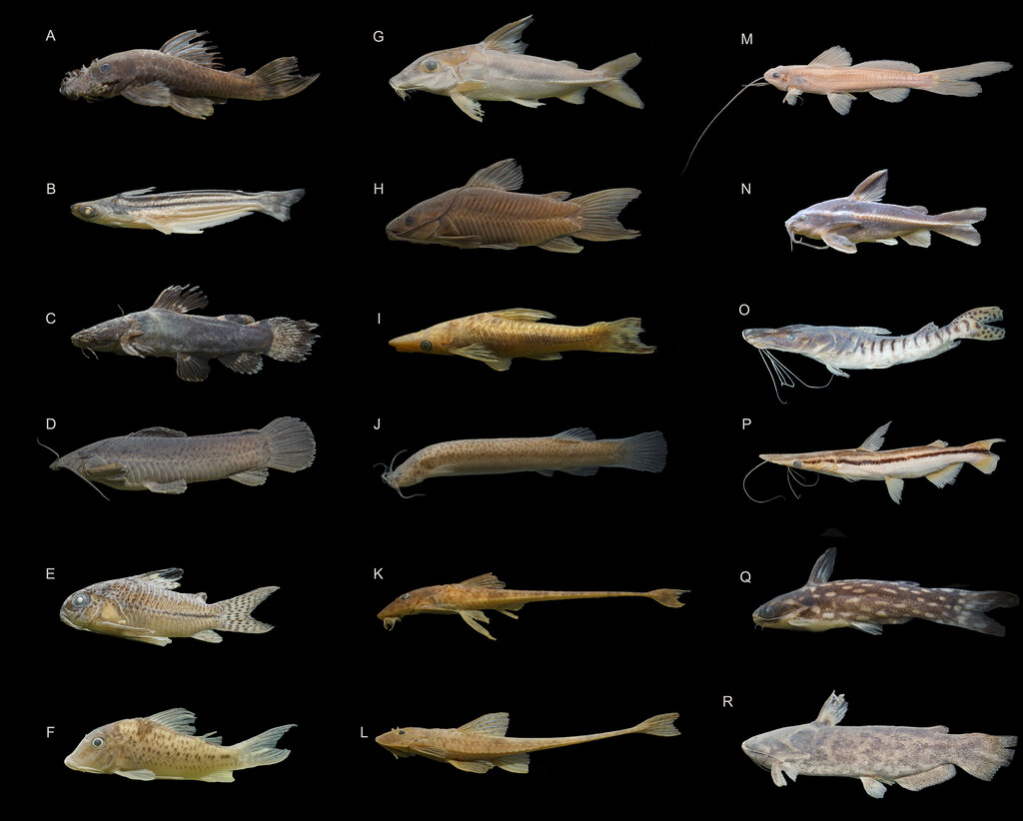
Selected fish species collected in the Munim River Basin of the Order Siluriformes: **A** *Ancistrus* sp. (CICCAA 06652, 76.85 mm SL), **B** *Auchenipterusmenezesi* (CICCAA 06534, 98.38 mm SL), **C** *Batrochoglanis* sp. (CICCAA 06654, 64.16 mm SL), **D** - *Callichthyscallichthys* (CICCAA 03927, 102.12 mm SL), **E** *Corydorasjulii* (CICCAA 06378, 34.33 mm SL), **F** *Corydorasvittatus* (CICCAA 06418, 34.19 mm SL), **G** *Hassaraffinis* (CICCAA 06263, 109.79 mm SL), **H** *Hoplosternumlittorale* (CICCAA 06657, 81.91 mm SL), **I** *Hypoptopomaincognitum* (CICCAA 06315, 70.81 mm SL), **J** Ituglaniscf.amazonicus (CICCAA 06643, 30.53 mm SL), **K** Loricariacf.cataphracta (CICCAA 06628, 105.80 mm SL), **L** *Loricariichthys* sp. (CICCAA 06328, 160.18 mm SL), **M** *Pimelodella* sp.1 (CICCAA 06629, 83.02 mm SL), **N** *Platydorasbrachylecis* (CICCAA 04608, 58.36 mm SL), **O** *Pseudoplatystomafasciatum* (CICCAA 04549, 208.39 mm SL), **P** *Sorubimlima* (CICCAA 06272, 204.01 mm SL), **Q** *Tatiaintermedia* (CICCAA 02736, 46.17 mm SL), **R** *Trachelyopterusgaleatus* (CICCAA 06243, 122.56 mm SL). Photographed by Lucas Vieira and Rafael Oliveira, edited by Axel Katz.

**Figure 5.**
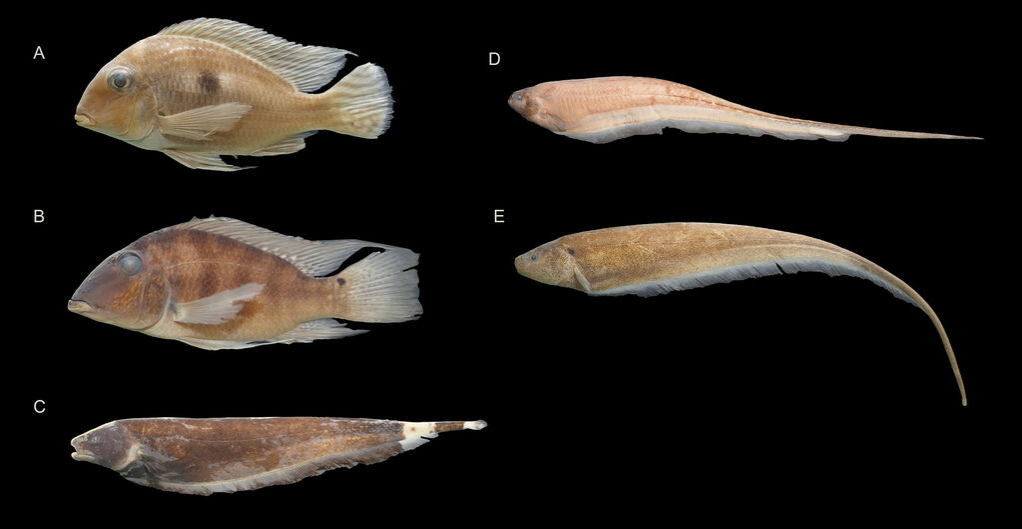
Selected fish species collected in the Munim River Basin of the Orders Cichliformes and Gymnotiformes: **A** *Geophagusparnaibae* (CICCAA 06229, 98.62 mm SL), **B** *Satanopercajurupari* (CICCAA 06377, 105.36 mm SL), **C** *Apteronotusalbifrons* (CICCAA 06266, 168.59 mm TL), **D** *Eigenmanniarobsoni* (CICCAA 06631, 180.36 mm TL), **E** *Sternopygusmacrurus* (CICCAA 06261, 183.50 mm TL). Photographed by Lucas Vieira and Rafael Oliveira, edited by Axel Katz.

Specimens of some species were photographed in the laboratory to illustrate the diversity of species that occur in the Munim River Basin (Fig. 3, Fig. 4 and Fig. 5). Additional photographs of Munim River fish species can be seen in Guimarães et al. (2018b): figs. 1, 2, Oliveira et al. (2020): fig. 3, Guimarães et al. (2021c) and Aguiar et al. (2022): fig.2b.

### Migratory species

Species were classified as migratory based on Carolsfeld et al. (2003). When any species was not listed in Carolsfeld et al. (2003), we considered the genus to indicate if it is a migratory species.

### General species accumulation curve

A matrix of occurrence and abundance data over the sampling period, for this study, was used to plot the general species accumulation curve with Primer-e statistical software (Clarke and Gorley 2006), based on a spreadsheet containing relevant data for this analysis (Suppl. material 2). Given that the order of samples in the analysis affects the shape of the curve produced, due to heterogeneity amongst the species in the samples (Ugland et al. 2003), 1000 permutations were calculated to overcome this effect.

### Species Richness and Fish assemblage composition

The statistical and ecological analyses were based on a spreadsheet containing relevant data for these analyses (Suppl. material 2).

**Species Richness**

Species richness (Sprich) (i.e. number of species in each river basin section) was compared using Kruskal-Wallis tests, on account of non-normal distribution (per Shapiro-Wilk test) and Dunn post-hocs with Holm adjusted p-values to account for multiple comparisons were used to determine section level differences. Visualisation was completed through the R package '“ggstatsplot'” (Patil 2021).

**Fish assemblage composition**

Fish assemblage composition was compared between basin sections, at the basin section level, using presence-absence data due to surveys not being standardised for sampling methods. Only native species were included in the analysis. First, nestedness was assessed using the NODF method (Almeida-Neto et al. 2008), which is bound between 0 and 100 where 100 is perfect nestedness, via vegan::nestednodf, then Sørensen dissimilarity and Simpsons Index were calculated using vegan::nestedbetasor. Sørensen dissimilarity closer to 0 indicates more shared species. Simpsons Index is not affected by species richness and represents true turnover, i.e. the the replacement of some species by other species from section to section, independent of potential differences in species richness between the sections. Areas with Simpsons Index values over 66% are considered to have similar faunal composition (Sánchez and López 1988). Jaccard Index was calculated using vegan::nestedbetajac where values closer to one indicate higher similarity. A cluster analysis and dendrogram was completed on the section Jaccard coefficients using the Ward.D2 method. All statistical analyses were performed within the R software environment version 4.0.2 and the package “vegan” (Oksanen et al. 2019, R Core Team 2020).

## Checklists

### Checklist of the fish fauna of the Munim River Basin

#### Actinopteri

BECF9992-741F-5B58-82A8-2E86F1450CC2

##### Notes

The checklist is presented in Table 2.

## Analysis

The present study recorded about 32500 specimens belonging to 123 fish species (94 identified at the species level) for the Munim River Basin, divided into 49 families and 14 orders (Table 2, Suppl. materials 1, 2). The most diverse orders are the Characiformes, with 43 species (35%); Siluriformes, with 38 species (30.9%); Acanthuriformes, with 11 (8.9%); Cichliformes, with seven species (5.7%) and Gymnotiformes, with six species (4.9%), representing 85.4% of all species known from the river basin. The remaining orders (Clupeiformes, Carangiformes, Cyprinodontiformes, Batrachoidiformes, Beloniformes, Mugiliformes, Scombriformes, Synbranchiformes and Tetraodontiformes) together represent only 14.6% of the Munim River Basin species.

The most diverse family was the Characidae, with 21 species (17.1%), followed by the Loricariidae, with nine (7.3%) and the Cichlidae, with seven (5.7%). Further, from all 123 recorded species, only two, *Oreochromisniloticus* and *Colossomamacropomum* are non-native species for the studied region and 13 are migratory species (see Table 2). Amongst the species identified at the species level, 16 are endemic to the hydrographic regions of Maranhão and Parnaíba *sensu* Hubert and Renno (2006) (Mrn and Prn, respectively).

**Figure 6.**
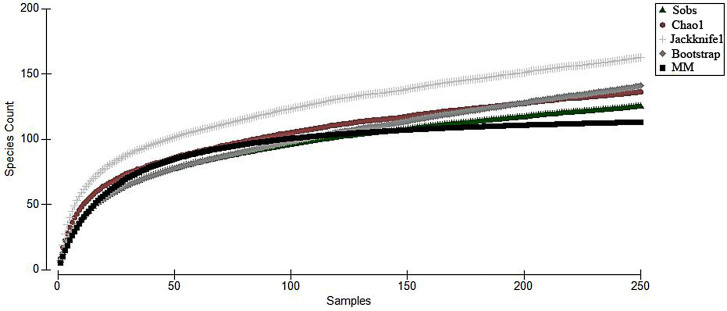
General species accumulation curve over the sampling period for this study.

According to the General plotted curves (General species accumulation curve), the sampling effort can be considered sufficient (Fig. 6), given that the observed values of *S*_obs_ (125 ±14) are aligned with those calculated in the estimator Chao1 (136.25) and the asymptote estimates of the Michaelis-Menten equation (113), as well as the Bootstrap (140.8) and Jackknife1 (162.85) variation indicators (Fig. 6).

**Species Richness**

**Figure 7.**
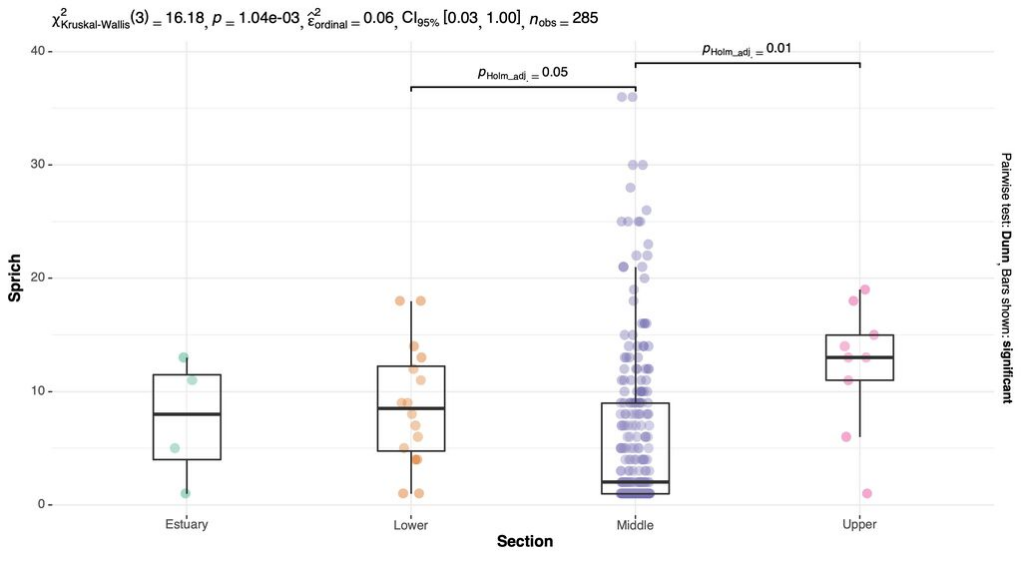
Species richness values for all sample sites across river basin sections.

There were significant differences in species richness between sections (*X^2^*= 16.207, df = 3, p < 0.001) where the Lower and Upper river basin sections had significantly more species than the Middle river basin section (p < 0.05, p < 0.01 respectively; Fig. 7).

**Fish assemblage composition**

**Figure 8.**
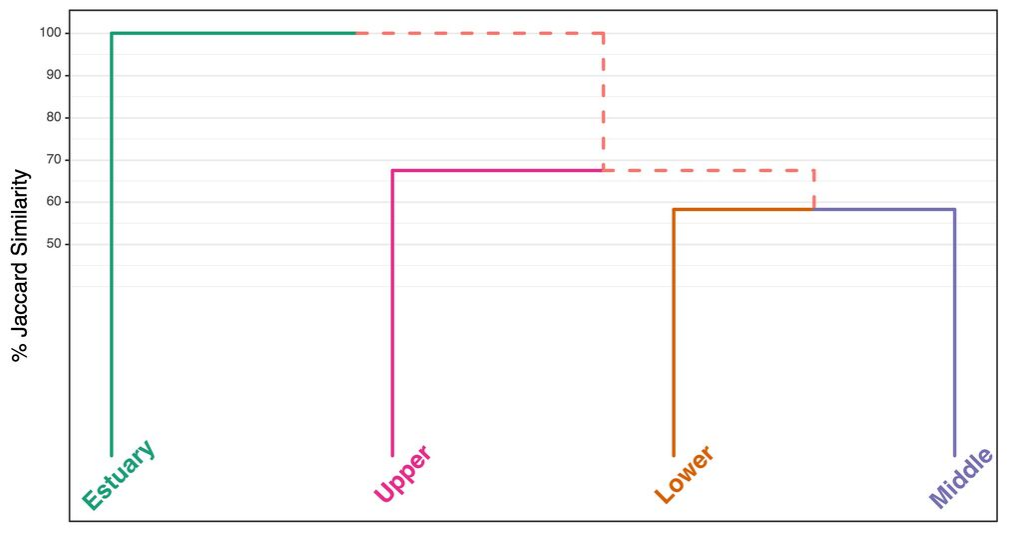
Hierachial cluster diagram of fish assemblage based on Jaccard Index per basin section using species presence-absence data.

There was weak nestedness across the four basin sections (NODF = 37.67) and indices of species composition similarity and dissimilarity were moderate. Where Sørensen dissimilarity was 0.70 and Simpsons Index (i.e. true turnover) was 56%, suggesting that fish assemblage is distinct between basin sections but only moderately. Jaccard similarity was 83% indicating many shared species compared to unique species across river basin sections. Cluster analysis showed that the Estuary and Upper river sections were more distinct from the Lower and Middle river sections, which formed their own cluster (Fig. 8).

## Discussion

This long-term icthyological survey, covering 12 years, conducted between 2010 and 2022 (65% of the surveys were carried out between 2019 and 2022), applied different sampling gears over different water bodies and environments along the Munim River Basin and recorded a predominance of fishes belonging to the Characiformes and Siluriformes, agreeing with a pattern expected for the Neotropics (Lowe-Mcconnell 1999, Pelicice et al. 2005, Langeani et al. 2007, Polaz et al. 2014, Reis et al. 2016, Brito et al. 2019, Dagosta and de Pinna 2019, Guimarães et al. 2020, Castro and Polaz 2020). The study also recorded the predominance of small-sized characid fishes, which have a great diversity in the Neotropical Region, due to several traits, such as their high trophic plasticity (Abelha et al. 2001, Van Der Sleen and Albert 2018, Dagosta and de Pinna 2019, Castro and Polaz 2020, Guimarães et al. 2020, Corrêa and Castro 2021).

A total of 123 species were recorded, with only two of them representing introduced species to the studied river basin (Table 2 and Suppl. materials 1, 2). *Colossomamacropomum* (tambaqui) occurs naturally in the Amazon and Orinoco River Basins, thus being native to Brazil, but not the Munim River Basin (Latini et al. 2016, Fricke et al. 2022b); and *Oreochromisniloticus* (tilápia) which is native to northern and eastern Africa (Figueredo and Giani 2005, Latini et al. 2016, Fricke et al. 2022b). All the other 121 species are native to the studied area. Therefore, the fish assemblage composition of the Munim River Basin is currently little affected by the presence of alien fish species. However, the policy regarding non-native species and push for economic development indicates this may soon change (Azevedo-Santos et al. 2011, Doria et al. 2021, Faria et al. 2022).

The occurrence of non-native fish species usually comes from fish farming and, in some cases, from intentional release and aquarium trade (Latini et al. 2016, Rocha et al. 2023). *Oreochromisnilotus* is an omnivorous fish which has broad abiotic tolerances, rapid growth and high survival in environments with high population density, traits which facilitate invasiveness and are favoured in aquaculture species (Figueredo and Giani 2005, Latini et al. 2016). In Brazil, the cultivation of this species is increasing, frequently without any control (Figueredo and Giani 2005, Latini et al. 2016). The species *C.macropomum* was recorded at only one collection site (a single specimen) (see Suppl. materials 1, 2). This makes us believe that the specimen had probably accidentally escaped from local or home fish-farming. On the other hand, *O.niloticus* was recorded in four locations (some of these locations far from each other), on different dates (i.e. several specimens). These data suggest establishment in the river basin and, thus, should be considered an established species in the Munim River Basin. Aquaculture initiatives with poor biosecurity are the probable pathway of invasion and rapid expansion facilitated by favourable climatic conditions should be expected and monitored in the Munim River Basin (Charvet et al. 2021, Wilgen et al. 2022). There is likelihood of negative ecological impacts as a result of this burgeoning invasion, in particular *O.niloticus* is a highly efficient filter feeder and may disrupt the food-web (Vasconcelos et al. 2018, Charvet et al. 2021). Biological invasions are a direct cause of biodiversity decline globally and are an increasing threat, especially in aquatic systems with high endemicity (Havel et al. 2015, Gallardo et al. 2016).

This study reported 13 migratory fish species occurring in the Munim River Basin. Therefore, the eventual construction of dams and hydroelectric plants will undoubtedly negatively impact these species as migration routes will be interrupted. Locally, Oliveira et al. (2020) have already reported this situation occurring in the Mata de Itamacaoca, Chapadinha Municipality, State of Maranhão. They verified that the reservoir dam constructed in the Mata de Itamacaoca inhibits the dispersion of fish occurring below the dam, which possesses higher species diversity. In addition, migratory species were also not found by Oliveira et al. (2020) above the dam, in the reservoir, which would be a suitable habitat for these species. This may illustrate the effects of increased dam construction along the Munim River Basin.

When comparing the present checklist with previous ones listing the fish species found in the hydrographic regions of Maranhão and Parnaíba *sensu* Hubert and Renno (2006) (Mrn and Prn, respectively) (e.g. Soares (2005), Barros et al. (2011), Nascimento et al. (2016), Piorski et al. (2017), Brito et al. (2019), Brito et al. (2020), Guimarães et al. (2020)), it is evident that the fish diversity from the Munim River Basin has been underestimated. In fact, the present study showed a surprisingly high fish species diversity occurring in the Munim River Basin, when compared to the species richness found in other larger drainage systems and river basins from Maranhão. For example, Munim River Basin outnumbered the Itapecuru River Basin, a larger river basin, with 29 more species being recorded, where 94 fish species are known to occur (e.g. Barros et al. (2011), Nascimento et al. (2016), Koerber et al. (2022)). In addition, we recorded 67 more species than in the Preguiças and Periá River Basin, where 56 fish species are known to occur (e.g. Piorski et al. (2017), Brito et al. (2019), Brito et al. (2020), Koerber et al. (2022)); 22 more fish species than Guimarães et al. (2020) recorded for the Pindaré River drainage (101 fish species); and more than twice the number of fish species for the coastal river basins of Gurupi, Maracaçumé, and Turiaçu, where less than 50 species are known for each of these river basins (Koerber et al. 2022). There are only three studies surveying Maranhão coastal drainage systems, which presented a higher number of species than this study. Ramos et al. (2014), who recorded 146 species for the Parnaíba River Basin and, later, Silva et al. (2015) provided an updated list with six additional species (152). Koerber et al. (2022) published a checklist of the freshwater species in Maranhão (CLOFFBR-MA), listing 136 species for the Mearim River Basin. The Munim River Basin had 13 fewer species than those recorded in the Mearim River Basin, one of the largest river basins in Maranhão and 29 fewer species than the Parnaíba River Basin, which is the largest hydrographic basin in north-eastern Brazil (Ramos et al. 2014, Silva et al. 2015, Koerber et al. 2022).

When analysing the present results in light of the previous surveys in the Munim River Basin, it is clear that all previous studies were geographically restricted to specific localities, extremely close to each other, thus were not able to depict and represent the wider basin diversity. Ribeiro et al. (2014) recorded only 20 fish species (103 less than the present study), using a traditional fishing technique called "moita" commonly used by local traditional communities in the Chapadinha Municipality. However, this method is biased toward the capture of medium to large-sized fishes and is generally applied by subsistence fisheries. Matavelli et al. (2015) surveyed the tadpoles occurring in lentic and lotic environments in Cerrado and Restinga vegetation types, sampling in localities in the Munim and Parnaíba river basins. Fish species were also sampled and a total of 13 species were recorded from the Munim River Basin (110 less than the present study). Nunes et al. (2019) carried out a weight-length ratio study of the fish community in one locality in Munim River Basin, recording 15 fish species (108 less than the present study). More recently, Oliveira et al. (2020) published a freshwater fish species list of a conservation unit in the Chapadinha Municipality after a long monitoring period, with 23 species (100 less than the present study). However, the survey was focused on small streams and consequently recorded mainly small-size species. Guimarães et al. (2021b) published a book from the same area studied by Oliveira et al. (2020), directed at the general public, which focused on species with an estimated high ornamental value. Finally, in the CLOFFBR-MA, which relied upon literature information, 59 species were identified in the Munim River Basin (64 less than the present study) (Koerber et al. 2022). None of these previous studies had the main goal of identifying the entire species diversity of the Munim River Basin.

Within the 121 native species listed in the present study, 29 were not able to be identified to the species level. Guimarães et al. (2018a) and Guimarães et al. (2020), hypothesised that probably this is a result of the lack of taxonomic knowledge and information about these species and groups occurring in Maranhão. The taxa which could not be identified to the species level, likely belong to species complexes or represent taxonomically challenging and poorly defined groups and may represent new species to science (see Table 2).

Median species richness in the Middle river basin section was lower than in the Upper and Lower river basin sections; however, the Middle section had both more sampling sites and much higher range of species richness. Environmental filtering across river gradients has a strong influence on species richness and diversity (López‐Delgado et al. 2019, Walsh et al. 2022). By grouping by section, we are missing local habitat-specific variables which are likely to be driving differences in fish assemblages across a highly heterogenous river network. Investigating habitat specific associations and drivers of beta diversity will vastly improve our understanding of drivers of fish assemblages in the Munim River Basin. Moderate nestedness and similarity/dissimilarity trends, combined with the lack of clear clustering between sites within river basin sections, indicate that fish assemblage structuring in the Munim River Basin is probably driven by both the river continuum concept as well as environmental filtering (Vannote et al. 1980, Heino et al. 2015). However, unobstructed flows facilitating dispersal likely drive high similarity throughout each basin section (Leitão et al. 2018). The Munim River Basin is not high altitude and has neither large rapids nor large waterfalls; therefore, the flow conditions through the sections are also relatively similar, with the lower river section differing through estuarine influence. Further research is needed to understand the specific microhabitats and fish associations throughout the river basin as this is undoubtedly a driving factor of diversity. For example, river slope and flow conditions exert strong environmental filters on fish community and traits in Neotropical and Afrotropical freshwaters and dispersal between heterogenous habitats may be limited by side channels and swamp habitats (Caetano et al. 2021, *Walsh et al. 2022*). A higher concentration of specialist species is expected to be found in the Upper section as there is more competition for niches (Sternberg and Kennard 2013). The cluster analysis indicated that the Upper section sites were on distinct branches from the other sites, but a standardised sampling methodology combined with implementation of functional trait-based approaches will facilitate our understanding of finer scale processes of environmental filtering in each section (Bower and Winemiller 2019a, Bower and Winemiller 2019b).

Considering all 92 native species which were identified to the species level, 30 of them (*Achirusachirus*, *Amphiariusrugispinis*, *Anablepsanableps*, *Anchoviasurinamensis*, *Anchoviellaguianensis*, *Anchoviellalepidentostole*, *Aspistorquadriscutis*, *Aspredoaspredo*, *Bagrebagre*, *Batrachoidessurinamensis*, *Cathoropsspixii*, *Centropomusparallelus*, *Chaetodipterusfaber*, *Chloroscombruschrysurus*, *Conodonnobilis*, *Cynoscionsteindachneri*, *Eugerresplumieri*, *Genyatremusluteus*, *Hyporhamphusroberti*, *Lutjanusjocu*, *Macrodonancylodon*, *Menticirrhusamericanus*, *Micropogoniasfurnieri*, *Mugilcurema*, *Oligoplitespalometa*, *Opisthonemaoglinum*, *Peprilusparu*, *Plagioscionsquamosissimus*, *Rhinosardiniaamazonica* and *Stellifernaso*) are commonly found in brackish water or estuaries. Due to this, no biogeographical considerations will be made about them. From the remaining 62 species identified to the species level, 16 are only known from river drainage systems and basins of the Maranhão State and the Parnaíba River Basin (*Anablepsoidesvieirai*, *Apistogrammapiauiensis*, *Auchenipterusmenezesi*, *Charaxawa*, *Cichlasomazarskei*, *Corydorasvittatus*, *Eigenmanniarobsoni*, *Geophagusparnaibae*, *Hassaraffinis*, *Hemiodusparnaguae*, *Hyphessobryconpiorskii*, *Pimelodellaparnahybae*, *Prochiloduslacustris*, *Rhamphichthysatlanticus*, *Roeboidesmargareteae* and *Roeboidessazimai*). Three other species (*Platydorasbrachylecis*, *Poeciliasarrafae* and *Schizodondissimilis*) are also known from other drainages in the northeast of Brazil (Teixeira et al. 2017, Silva et al. 2020, Fricke et al. 2022b). The remaining 43 species are also known from different Amazonian drainage systems (Fricke et al. 2022b), a pattern clearly showing the influence and presence of Amazonian fauna in the Munim River Basin. In addition, when comparing the species listed for the Munim River Basin to the list of species in the Parnaíba River (Ramos et al. 2014, Silva et al. 2015), there are 53 native species co-occurring in both drainage systems, showing a high influence of the larger Parnaíba River Basin over smaller coastal drainage systems. Finally, there are a total of 64 new records of fish species for the Munim River Basin and 48 new records considering only the number of taxa identified at the species level (Table 2), showing that, until the present study, the drainage's diversity was underestimated.

The Munim River Basin, previously a neglected river system, similar to many other coastal systems in Maranhão, is now one of the better known river basins relative to its fish diversity. A detailed taxonomic investigation of specimens sampled over a 12 year period revealed a much diverse fish fauna. The present study is the most comprehensive carried out in the Munim River Basin so far, adding 64 species (including species identified at the species level and species not identified at species level), which were previously considered not to occur in the drainage, resulting in a total of 123 species. Within this species richness, there was a large number of taxa, which could not be identified at the species level, indicating the urgent need for dedicated taxonomic research in the region. This study puts emphasis on the importance of compiling ichthyofaunal lists for poorly-studied or subsampled areas. This achievement represents a first step in understanding the diversity in the Munim River Basin, with the information presented herein allowing the development of future ecology, biogeography and conservation studies. Thus, this is an essential contribution to the effort to better understand the fish diversity of Maranhão in the face of rapid global change and habitat alteration. Despite the high number of species found for the Munim River Basin, more collection efforts are recommended, especially in the Lower and Estuary sections. New collection expeditions may find species that may not have been recorded by this work.

## Supplementary Material

XML Treatment for Actinopteri

9AE72826-4BE9-54A6-9F2A-6F6637B4E7C5 10.3897/BDJ.11.e98632.suppl1 Supplementary material 1 Checklist of the fish fauna of the Munim River Basin, Maranhão, north-eastern Brazil Data type Excel csv spreadsheet Brief description Spreadsheet in Darwin Core format. File: oo_805945.csv https://binary.pensoft.net/file/805945 Lucas Vieira

D4E1FA45-25A6-5F75-BC93-22566C2DE86A 10.3897/BDJ.11.e98632.suppl2 Supplementary material 2 Checklist of the fish fauna of the Munim River Basin, Maranhão, north-eastern Brazil Data type Excel csv spreadsheet Brief description Spreadsheet used in ecological and statistics analyses. File: oo_805830.csv https://binary.pensoft.net/file/805830 Lucas Vieira

## Figures and Tables

**Table 1.** Sampling sites at the Munim River Basin, Maranhão, Brazil. *Localities with the presence of non-native species.

**Locality number (L)**	**Locality**	**Municipality**	**Coordinates**	**Altitude**	**River basin section**
01	Stream in the balneário at the entrance of Anapurus	Anapurus - MA	03°40'15.28"S 043°07'9.7"W	81 m	Middle
02	*Stream at balneário São Lourenço	Anapurus - MA	03°39'16.30"S 043°6'50.2"W	75 m	Middle
03	Stream at balneário Recanto do Buriti	Anapurus - MA	03°40'53.04"S 043°7'23.0"W	76 m	Middle
04	Riacho crossing the road at Poços community	Anapurus - MA	03°33'44.61"S 043°3'52.4"W	71 m	Middle
05	Stream at Caraíbas community	Chapadinha - MA	03°56'7.71"S 043°26'14.8"W	51 m	Middle
06	Riacho Xororó at Aparecida neighbourhood	Chapadinha - MA	03°44'2.23"S 043°22'1.21"W	81 m	Middle
07	Stream at Aldeia neighbourhood	Chapadinha - MA	03°45'7.75"S 043°21'32.7"W	74 m	Middle
08	Stream at Aldeia neighbourhood	Chapadinha - MA	03°44'53.1"S 043°21'32.6"W	80 m	Middle
09	Stream at Terra Duras neighbourhood	Chapadinha - MA	03°45'6.42"S 043°22'24.7"W	65 m	Middle
10	Riacho Feio, Boa Vista community	Chapadinha - MA	03°50'51.8"S 043°18'50.5"W	44 m	Middle
11	*Riacho Feio, Boa Vista community	Chapadinha - MA	03°50'46.8"S 043°18'48.9"W	40 m	Middle
12	Riacho Feio, São José community	Chapadinha - MA	03°51'6.30"S 043°17'53.0"W	45 m	Middle
13	*Riacho Feio, São José community	Chapadinha - MA	03°51'18.7"S 043°17'14.4"W	47 m	Middle
14	Riachinho, Cumbre community	Chapadinha - MA	03°51'46.8"S 043°17'10.2"W	52 m	Middle
15	Riachinho, Água Branca community	Chapadinha - MA	03°53'13.5"S 043°16'37.1"W	59 m	Middle
16	Riacho Feio, Riacho Feio community	Chapadinha - MA	03°51'42.84"S 043°16'1.7"W	52 m	Middle
17	Rio Iguará, Malhadinha community	Vargem Grande - MA	03°54'27.8"S 043°44'55.8"W	30 m	Middle
18	Rio Iguará, Malhadinha community	Vargem Grande - MA	03°54'3.25"S 043°44'55.8"W	32 m	Middle
19	Rio Iguará, Poço Cumprido community	Chapadinha - MA	04°2'54.24"S 043°34'58.4"W	41 m	Middle
20	Stream at Itamacaoca forest	Chapadinha - MA	03°44'45.2"S 043°19'15.0"W	90 m	Middle
21	Stream at balneário Repouso do Guerreiro	Chapadinha - MA	03°44'57.4"S 043°20'24.0"W	66 m	Middle
22	Stream at Itamacaoca forest	Chapadinha - MA	03°44'27.2"S 043°19'36.5"W	85 m	Middle
23	Itamacaoca dam	Chapadinha - MA	03°44'56.5"S 043°19'55.8"W	74 m	Middle
24	Stream just after Itamacaoca dam	Chapadinha - MA	03°45'7.42"S 043°20'4.05"W	68 m	Middle
25	Jabuti community, Tinguis road	Chapadinha - MA	03°46'11.9"S 043°20'25.2"W	50 m	Middle
26	Rio Preto at Bom Sucesso community	Mata Roma - MA	03°34'0.40"S 043°19'0.40"W	45 m	Middle
27	Swampy areas at Brejo do Meio community	Chapadinha - MA	03°55'38.7"S 043°30'13.1"W	53 m	Middle
28	Stream behind the Mix Atacarejo Mateus store	Chapadinha - MA	03°45'6.00"S 043°20'23.0"W	59 m	Middle
29	Rio Munim, Carnaúba Amarela community	Chapadinha - MA	03°51'51.3"S 043°19'36.8"W	39 m	Middle
30	Rio Munim, Porções bridge	Chapadinha - MA	03°50'50.0"S 043°19'19.4"W	41 m	Middle
31	*Rio Munim, Cedro community	Chapadinha - MA	03°50'15.5"S 043°19'41.1"W	41 m	Middle
32	Rio Munim, Riacho Fundo community	Chapadinha - MA	03°42'22.7"S 043°31'47.1"W	25 m	Middle
33	Rio Munim, bridge at Mangabeira community	Chapadinha - MA	03°48'34.1"S 043°24'33.2"W	33 m	Middle
34	Stream at Pai Gonçalo community	Chapadinha - MA	04°2'38.12"S 043°28'40.7"W	82 m	Middle
35	Stream at Mucambo community	São Benedito do Rio Preto - MA	03°29'1.01"S 043°33'39.5"W	92 m	Middle
36	Riacho da Raiz	Chapadinha - MA	03°53'45.1"S 043°29'21.3"W	45 m	Middle
37	Riacho São João, São João dos Pilão	Brejo - MA	03°41'2.64"S 042°56'31.9"W	89 m	Middle
38	Riacho Pau Preto, Pau Preto community	Brejo - MA	03°39'54.9"S 042°56'35.5"W	84 m	Middle
39	Riacho da Cruz, close to Palestina	Brejo - MA	03°41'18.0"S 042°58'39.8"W	88 m	Middle
40	Rio Preto, Água Rica community	Brejo - MA	03°41'34.92"S 043°0'56.1"W	78 m	Middle
41	Córrego Água Rica	Anapurus - MA	03°41'41.24"S 043°1'44.8"W	86 m	Middle
42	Riacho do Muquém	Mata Roma - MA	03°42'21.2"S 043°13'57.1"W	68 m	Middle
43	Stream, Laranjeira community	Buriti - MA	03°52'31.09"S 043°3'0.60"W	96 m	Middle
44	Rio Munim, Capoeira Grande community	Afonso Cunha - MA	04°10'2.79"S 043°13'28.0"W	54 m	Upper
45	Rio São Gonçalo	Afonso Cunha - MA	04°7'58.77"S 043°19'16.1"W	64 m	Upper
46	Stream crossing the road	Afonso Cunha - MA	04°10'53.63"S 043°14'1.5"W	58 m	Upper
47	Stream crossing the road	Afonso Cunha - MA	04°12'23.9"S 043°14'46.8"W	68 m	Upper
48	Riacho barrigudinho	Afonso Cunha - MA	04°18'46.1"S 043°13'39.1"W	67 m	Upper
49	Riacho do boi	Afonso Cunha - MA	04°19'12.38"S 043°13'9.8"W	67 m	Upper
50	Stream crossing the road	Aldeias Altas - MA	04°22'14.3"S 043°12'17.6"W	69 m	Upper
51	Riacho do boi	Aldeias Altas - MA	04°26'4.96"S 043°11'46.9"W	82 m	Upper
52	*Rio Bandeira, Belágua	Belágua - MA	03° 9'22.7"S 043°30'35.4"W	65 m	Middle
53	Riacho Água Fria on the road MA-110	Belágua - MA	03°10'9.49"S 043°28'45.3"W	68 m	Middle
54	Rio Bandeira	Urbano Santos - MA	03°11'49.0"S 043°24'29.3"W	41 m	Middle
55	Rio Mocambo	Urbano Santos - MA	03°12'34.6"S 043°24'23.8"W	38 m	Middle
56	Stream on the road MA-224	São Benedito do Rio Preto - MA	03°18'46.2"S 043°30'25.1"W	40 m	Middle
57	Rio Preto, São Benedito do Rio Preto	São Benedito do Rio Preto - MA	03°19'59.0"S 043°31'34.8"W	29 m	Middle
58	Stream on the road MA-224	São Benedito do Rio Preto - MA	03°29'29.0"S 043°35'25.9"W	50 m	Middle
59	Rio Munim, on the road MA-224	Nina Rodrigues - MA	03°35'14.1"S 043°39'50.4"W	21 m	Middle
60	Rio Munim, at the quilombola community Evienã	Presidente Vargas - MA	03°22'31.0"S 043°58'18.5"W	14 m	Middle
61	Riacho Paulica on the road MA-020	Presidente Vargas - MA	03°25'54.98"S 043°58'1.0"W	16 m	Middle
62	Rio Munim at Nina Rodrigues City	Nina Rodrigues - MA	03°27'36.1"S 043°54'15.1"W	14 m	Middle
63	Riacho Paulica on the road BR-222	Vargem Grande -MA	03°31'11.5"S 043°58'30.7"W	23 m	Middle
64	Rio Iguará on the road BR-222	Vargem Grande - MA	03°33'9.64"S 043°52'23.0"W	22 m	Middle
65	Rio Munim mouth at Icatu	Icatu - MA	02°46'33.86"S 044° 4'1.3"W	1 m	Estuary
66	Rio Una, between the municipalities of Morro and Icatu	Morros - MA	02°50'3.06"S 044°2'24.82"W	8 m	Lower
67	Rio das Cobra, Santa Helena community	Morros - MA	02°49'22.1"S 044° 2'34.8"W	9 m	Lower
68	Riacho at the entrance to Icatu	Icatu - MA	02°46'58.50"S 044°2'48.2"W	19 m	Lower
69	Rio Munim, Cachoeira Grande	Cachoeira Grande - MA	02°55'36.25"S 044°3'39.2"W	4 m	Lower
70	Stream crossing the road MA-020	Cachoeira Grande - MA	02°55'14.62"S 044°2'31.5"W	34 m	Lower
71	Stream next to the road MA-402	Axixá - MA	02°51'37.1"S 044° 3'14.5"W	4 m	Lower
72	Rio Munim between the municipalities of Axixá and Presidente Juscelino	Axixá - MA	02°52'35.63"S 044°3'41.8"W	15 m	Lower
73	Stream between the municipalities of Axixá and Presidente Juscelino	Axixá - MA	02°53'50.06"S 044°4'15.9"W	4 m	Lower
74	Rio Munim, Presidente Juscelino	Presidente Juscelino - MA	02°55'39.38"S 044°3'50.5"W	6 m	Lower
75	Rio Una, Cachoeira do Arruda	Morros - MA	02°53'31.5"S 043°58'13.8"W	28 m	Lower
76	Riacho das Pacas	Morros - MA	02°51'4.94"S 043°57'52.1"W	28 m	Lower
77	Stream next to the road MA-402	Morros - MA	02°51'19.5''S 044°01'03.0''W	19 m	Lower
78	Rio Munim, Axixá	Axixá - MA	02°50'14.60"S 044°3'3.81"W	1 m	Lower
79	Rio Munim, Balceiro community	Chapadinha - MA	03°46'44.9"S 043°26'42.7"W	33 m	Middle
80	Stream at the Paiol community	Chapadinha - MA	04°1'13.56"S 043°29'27.6"W	74 m	Middle
81	Stream at São Pedro community	Chapadinha - MA	03°54'4.66"S 043°35'12.3"W	73 m	Middle
82	Stream crossing a road in the Resex	Chapadinha - MA	03°56'10.0"S 043°30'29.5"W	61 m	Middle
83	Riacho Xororó at Aparecida neighbourhood	Chapadinha - MA	03°44'7.77"S 043°22'8.94"W	69 m	Middle
84	Riachinho, Água Branca community	Chapadinha - MA	03°52'37.67"S 043°16'59.37"W	60 m	Middle

**Table 2.** List of fish species recorded for the Munim River Basin in the present study. *endemic species to the hydrological units Maranhão and Parnaíba *sensu* Hubert and Renno (2006).

**CLASS/ORDER/FAMILY/SPECIES**	**New records**	**Migratory species**	**Non-native species**	**Habitat of occurrence**	**Common name (Portuguese)**
CLASS ACTINOPTERI					
**ACANTHURIFORMES**					
**Ephippidae**					
*Chaetodipterusfaber* (Broussonet, 1782)	X			Marine, Estuary and Freshwater	Peixe enxada
**Gerreidae**					
*Eugerresplumieri* (Cuvier, 1830)	X			Marine, Estuary and Freshwater	Mojarra
**Haemulidae**					
*Conodonnobilis* (Linnaeus, 1758)	X			Marine, Estuary and Freshwater	
*Genyatremusluteus* (Bloch, 1790)	X			Marine and Estuary	
**Lutjanidae**					
*Lutjanusjocu* (Bloch & Schneider, 1801)	X			Marine, Estuary and Freshwater	
**Sciaenidae**					
*Cynoscionsteindachneri* (Jordan, 1889)	X			Marine, Estuary and Freshwater	
*Macrodonancylodon* (Bloch & Schneider, 1801)	X			Marine and Estuary	
*Menticirrhusamericanus* (Linnaeus, 1758)	X			Marine and Estuary	
*Micropogoniasfurnieri* (Desmarest, 1823)	X			Marine, Estuary and Freshwater	Curvina
*Plagioscionsquamosissimus* (Heckel, 1840)	X	X		Freshwater	Curvina
*Stellifernaso* (Jordan, 1889)	X			Estuary and Freshwater	
**BATRACHOIDIFORMES**					
**Batrachoididae**					
*Batrachoidessurinamensis* (Bloch & Schneider, 1801)	X			Marine and Estuary	Pacamão
**BELONIFORMES**					
**Hemiramphidae**					
*Hyporhamphusroberti* (Valenciennes, 1847)	X			Marine and Estuary	Agulha
**CARANGIFORMES**					
**Achiridae**					
*Achirusachirus* (Linnaeus, 1758)	X			Marine, Estuary and Freshwater	Linguado
**Carangidae**					
*Chloroscombruschrysurus* (Linnaeus, 1766)	X			Marine and Estuary	Palombeta
*Oligoplitespalometa* (Cuvier, 1832)	X			Marine, Estuary and Freshwater	Tibiro
**Centropomidae**					
*Centropomusparallelus* Poey, 1860	X			Marine, Estuary and Freshwater	Robalo
**CHARACIFORMES**					
**Acestrorhynchidae**					
*Acestrorhynchusfalcatus* (Bloch 1794)				Freshwater	Lubarana
**Anostomidae**					
Leporinusaff.friderici		X		Freshwater	Piau de coco
*Schizodondissimilis* (Garman 1890)		X		Freshwater	Piau de vara
**Characidae**					
*Aphyocharax* sp.				Freshwater	Enfermerinha
Astyanaxcf.bimaculatus				Freshwater	Piaba rabo de fogo
*Brachychalcinusparnaibae* Reis 1989	X			Freshwater	Piaba chatinha
*Charaxawa Guimarães*, Brito, Ferreira & Ottoni, 2018*				Freshwater	Cacunda
Ctenobryconcf.spilurus				Freshwater	Piaba
*Hemigrammus* sp. 1 *sensu* Oliveira et al. (2020)				Freshwater	Piaba
*Hemigrammus* sp.2 *sensu* Oliveira et al. (2020)				Freshwater	Piaba
Hemigrammuscf.rodwayi				Freshwater	Piaba
*Hyphessobryconpiorskii* Guimarães, Brito, Feitosa, Carvalho-Costa & Ottoni, 2018*				Freshwater	Tetra
*Knodusguajajara* Aguiar, Brito, Ottoni & Guimarães, 2022*				Freshwater	Piaba
*Microschemobrycon* sp.				Freshwater	Piaba
*Moenkhausia* sp.				Freshwater	Piaba
Moenkhausiacf.intermedia				Freshwater	Piaba
*Moenkhausiaoligolepis* (Günther, 1864)				Freshwater	Piaba rabo preto
Phenacogastercf.pectinata				Freshwater	Lambarizinho
*Poptellacompressa* (Günther, 1864)				Freshwater	Piaba chatinha
*Psellogrammuskennedyi* (Eigenmann, 1903)	X			Freshwater	
*Roeboidesmargareteae* Lucena, 2003*				Freshwater	Cacunda
*Roeboidessazimai* Lucena, 2007*				Freshwater	Cacunda
*Serrapinnus* sp.				Freshwater	Piabinha
*Tetragonopterusargenteus* Cuvier 1816	X			Freshwater	Piaba
**Crenuchidae**					
*Characidium* sp.				Freshwater	Canivete, mocinha
**Curimatidae**					
Curimatopsisaff.cryptica				Freshwater	
*Psectrogasterrhomboides* Eigenmann & Eigenmann 1889				Freshwater	Branquinha
*Steindachnerinanotonota* (Miranda Ribeiro, 1937)				Freshwater	João duro
**Cynodontidae**					
*Cynodongibbus* (Agassiz, 1829)				Freshwater	Gata
**Erythrinidae**					
*Hopliasmalabaricus* (Bloch, 1794)				Freshwater	Traíra
*Hoplerythrinusunitaeniatus* (Spix & Agassiz, 1829)				Freshwater	Iú
**Gasteropelecidae**					
*Gasteropelecussternicla* (Linnaeus, 1758)				Freshwater	Borboleta
**Hemiodontidae**					
*Hemiodusparnaguae* Eigenmann & Henn, 1916*				Freshwater	Flecheiro
**Iguanodectidae**					
Bryconopsaff.affinis				Freshwater	Dórico
**Lebiasinidae**					
*Copellaarnoldi* (Regan, 1912)				Freshwater	
*Nannostomusbeckfordi* Günther, 1872				Freshwater	Peixe lápis
**Triportheidae**					
*Triportheussignatus* (Garman, 1890)		X		Freshwater	Sardinha de água doce
**Prochilodontidae**					
*Prochiloduslacustris* Steindachner, 1907*		X		Freshwater	Curimatá
**Serrasalmidae**					
*Colossomamacropomum* (Cuvier, 1816)	X	X	X	Freshwater	Tambaqui
*Metynnislippincottianus* (Cope, 1870)				Freshwater	Pacú
*Myloplusrubripinnis* (Müller & Troschel, 1844)	X			Freshwater	Pacú folha
*Serrasalmusrhombeus* (Linnaeus, 1766)	X	X		Freshwater	Pirambeba
*Pygocentrusnattereri* Kner, 1858		X		Freshwater	Piranha vermelha
**CICHLIFORMES**					
**Cichlidae**					
*Aequidenstetramerus* (Heckel, 1840)				Freshwater	Cará, Acará
*Apistogrammapiauiensis* Kullander, 1980*				Freshwater	Carazinho
*Cichlasomazarskei* Ottoni, 2011*				Freshwater	Cará preto, Acará, Cará
*Crenicichlabrasiliensis* (Bloch, 1792)				Freshwater	Lope, Joana, Sabão
*Geophagusparnaibae* Staeck & Schindler, 2006*				Freshwater	Cará
*Oreochromisniloticus* (Linnaeus, 1758)	X		X	Estuary and Freshwater	Tilápia do nilo
*Satanopercajurupari* (Heckel, 1840)				Freshwater	Cará bicudo
**CLUPEIFORMES**					
**Engraulidae**					
*Anchoviasurinamensis* (Bleeker, 1865)	X			Estuary and Freshwater	Manjuba
*Anchoviellaguianensis* (Eigenmann, 1912)	X			Estuary and Freshwater	Manjuba
*Anchoviellalepidentostole* (Fowler, 1911)	X			Marine, Estuary and Freshwater	Manjuba
**Clupeidae**					
*Opisthonemaoglinum* (Lesueur, 1818)	X			Marine and Estuary	Sardinha
*Rhinosardiniaamazonica* (Steindachner, 1879)	X			Estuary and Freshwater	Sardinha
**CYPRINODONTIFORMES**					
**Anablepidae**					
*Anablepsanableps* (Linnaeus, 1758)	X			Estuary and Freshwater	Tralhoto
**Poeciliidae**					
*Poeciliasarrafae* Bragança & Costa, 2011				Freshwater	Barrigudinho
**Rivulidae**					
*Anablepsoidesvieirai* Nelson, 2016*				Freshwater	Peixe de poça
**GYMNOTIFORMES**					
**Apteronotidae**					
*Apteronotusalbifrons* (Linnaeus, 1766)				Freshwater	Sarapó, Catana
**Gymnotidae**					
*Gymnotuscarapo* Linnaeus, 1758				Freshwater	Sarapó, Catana
**Hypopomidae**					
*Brachyhypopomus* sp.				Freshwater	Sarapó, Catana
**Sternopygidae**					
*Eigenmanniarobsoni* Dutra, Ramos & Menezes 2022*				Freshwater	Sarapó, Catana
*Sternopygusmacrurus* (Bloch & Schneider, 1801)				Freshwater	Sarapó, Catana
**Rhamphichthyidae**					
*Rhamphichthysatlanticus* Triques, 1999*				Freshwater	Tubiba, Sarapó
**MUGILIFORMES**					
**Mugilidae**					
*Mugilcurema* Valenciennes, 1836				Marine, Estuary and Freshwater	Sardinha
**SILURIFORMES**					
**Ariidae**					
*Amphiariusrugispinis* (Valenciennes, 1840)	X			Marine and Estuary	Bagre
*Aspistorquadriscutis* (Valenciennes, 1840)	X			Marine, Estuary and Freshwater	Bagre
*Bagrebagre* (Linnaeus, 1766)	X			Marine and Estuary	Bagre
*Cathoropsspixii* (Agassiz, 1829)	X			Marine, Estuary and Freshwater	Bagre
**Aspredinidae**					
*Aspredoaspredo* (Linnaeus, 1758)	X			Marine, Estuary and Freshwater	Banjo catfish
*Pseudobunocephalustimbira* Leão, Carvalho, Reis & Wosiacki, 2019	X			Freshwater	
**Auchenipteridae**					
*Auchenipterusmenezesi* Ferraris & Vari, 1999*	X			Freshwater	Bagre
*Tatiaintermedia* (Steindachner, 1877)	X			Freshwater	Bagrinho
*Trachelyopterusgaleatus* (Linnaeus, 1766)				Freshwater	Cangati, Bagrinho
**Callichthyidae**					
Aspidorascf.raimundi				Freshwater	Cari
*Callichthyscallichthys* (Linnaeus, 1758)				Freshwater	Cascudo
*Corydorasjulii* Steindachner, 1906	X			Freshwater	Cari
*Corydorasvittatus* Nijssen, 1971*	X			Freshwater	Cari
*Hoplosternumlittorale* (Hancock, 1828)	X			Freshwater	Cascudo
*Megalechisthoracata* (Valenciennes, 1840)				Freshwater	Cascudo
**Doradidae**					
*Hassaraffinis* (Steindachner, 1881)*				Freshwater	Cabeça de cavalo
*Platydorasbrachylecis* Piorski, Garavello, Arce H. & Sabaj Pérez, 2008	X			Freshwater	Guirri
**Loricariidae**					
Ancistruscf.damasceni				Freshwater	Mão na cara, Cascudo, Bodó
*Ancistrus* sp.				Freshwater	Mão na cara, Cascudo, Bodó
*Hemiodontichthysacipenserinus* (Kner, 1853)				Freshwater	Cachimbo
Hypostomuscf.krikati				Freshwater	Boi de carro, Cascudo, Bodó
*Hypostomus* sp.				Freshwater	Boi de carro, Cascudo, Bodó
*Hypoptopomaincognitum* Aquino & Schaefer, 2010	X			Freshwater	Cascudo
Loricariacf.cataphracta				Estuary and Freshwater	Cachimbo, Cascudo
*Loricariichthysderbyi* Fowler, 1915	X			Freshwater	Cachimbo, Cascudo
*Rineloricaria* sp.				Freshwater	Cachimbo, Cascudo
**Heptapteridae**					
*Imparfinis* sp.				Freshwater	Mandi
*Pimelodellaparnahybae* Fowler, 1941*				Freshwater	Mandi
*Pimelodella* sp1.				Freshwater	Mandi
*Pimelodella* sp2.				Freshwater	Mandi
*Rhamdiaquelen* (Quoy & Gaimard, 1824)	X			Freshwater	Jundiá
**Pimelodidae**					
*Hemisorubimplatyrhynchos* (Valenciennes, 1840)		X		Freshwater	Mandi três pinta
*Pimelodusblochii* Valenciennes, 1840	X	X		Estuary and Freshwater	Mandi
*Pimelodusornatus* Kner, 1858		X		Freshwater	Mandi dourado
*Pseudoplatystomafasciatum* (Linnaeus, 1766)	X	X		Freshwater	Surubim
*Sorubimlima* (Bloch & Schneider, 1801)		X		Freshwater	Bico de pato
**Pseudopimelodidae**					
*Batrochoglanis* sp.				Freshwater	
**Trichomycteridae**					
Ituglaniscf.amazonicus				Freshwater	
**SCOMBRIFORMES**					
**Stromateidae**					
*Peprilusparu* (Linnaeus, 1758)	X			Marine and Estuary	
**SYNBRANCHIFORMES**					
**Synbranchidae**					
*Synbranchusmarmoratus* Bloch 1795				Freshwater and Estuary	Muçum
**TETRAODONTIFORMES**					
**Tetraodontidae**					
Lagocephaluscf.lagocephalus	X			Marine and Estuary	Baiacu arara
